# Evaluating the performance of a new ergonomic laparoscopic needle holder for intracorporeal suturing

**DOI:** 10.1371/journal.pone.0313568

**Published:** 2025-01-24

**Authors:** Arturo Minor-Martínez, Ricardo Manuel Ordorica-Flores, Juan Ramón Mota-Carmona, Iván Tlacaélel Franco-González, Jesús Tapia Jurado, Fernando Pérez-Escamirosa

**Affiliations:** 1 Departamento de Ingeniería Eléctrica, Sección de Bioelectrónica, Centro de Investigación y de Estudios Avanzados del Instituto Politécnico Nacional (CINVESTAV–IPN), Ciudad de México, México; 2 Servicio de Cirugía Endoscópica, Hospital Infantil de México Federico Gómez, Ciudad de México, México; 3 Departamento de Cirugía, Facultad de Medicina, Universidad Nacional Autónoma de México (UNAM), Ciudad de México, México; 4 Instituto de Ciencias Aplicadas y Tecnología (ICAT), Universidad Nacional Autónoma de México (UNAM), Ciudad de México, México; Tokyo Women’s Medical University, JAPAN

## Abstract

In laparoscopy, the absence of ergonomics in the instruments affects the performance and efficiency of the surgeon, increasing the likelihood of developing musculoskeletal injuries. This article presents the development of a laparoscopic needle holder with an ergonomic handle and the experience in its use with surgeons in the intracorporeal suturing task. The handle of the laparoscopic needle holder consists of a semi-spherical piece that easily adapts to the palm of the surgeon’s hand and improves the posture and ergonomics of the wrist, allowing the direct transmission of rotational movements around the longitudinal axis of the instrument towards the tip. Moreover, a spring-loaded mechanism allows the tip of the tool to be opened and closed in a normally closed configuration, with enough pressure to hold different surgical needles. Twenty-two pediatric surgery surgeons and residents, without upper extremity musculoskeletal disorders, participated in this study. Each participant performed the intracorporeal suturing task using two laparoscopic needle holders (conventional and with ergonomic handle) in a laparoscopic simulator. Motion data from both instruments were captured and the performance was assessed by means of 16 motion analysis parameters (MAPs). The performance of the residents improved markedly using the laparoscopic needle holder with ergonomic handle, obtaining statistically significant differences in 10 MAPs evaluated. The comparison of the results of each instrument showed that this ergonomic laparoscopic needle holder stood out over the conventional tool, improving psychomotor skills and the ability to control the angular position and rotational movements of the instrument on its own axis, indispensable for intracorporeal suturing. The innovative design of the laparoscopic needle holder with ergonomic handle allows for better transfer of rotational movements from the surgeon’s hand directly with the longitudinal axis to the tip, reducing extreme wrist positions and excessive effort to open and close the tip of the instrument during intracorporeal suturing.

## 1. Introduction

One of the significant hurdles in laparoscopic surgery lies in the intricacies of intracorporeal knot suturing. Mastering the technique of intracorporeal suturing stands as a crucial and indispensable skill that surgeons must acquire and refine to be deemed experienced in its execution [1, 2]. As the complexity of various laparoscopic procedures increases, there are mechanical and ergonomic limitations in the surgical instruments that curtail manual dexterity and affect a surgeon’s performance in addressing these surgical challenges [3].

One of the complexities inherent in laparoscopic surgery is the procedure being conducted through a small incision point, serving as a pivot in two perpendicular planes, restricting the surgical instrument’s movement to only 4 degrees of freedom (DoF). This incision point generates a phenomenon known as the ‘fulcrum effect’, altering the movements of the instrument’s tip concerning the handle during surgery [4]. As a result, surgeons assume rigid postures within the operating room, creating muscular tension in their limbs for prolonged periods, leading to discomfort, fatigue, as well as mental stress and frustration due to these limitations [5, 6]. Additionally, the absence of integrated ergonomic considerations in laparoscopic instruments could lead to the onset of cumulative trauma disorders, increasing the probability of developing musculoskeletal injuries and physical repercussions in the surgeon’s body in the medium and long term [6–8].

To address these perceived constraints in laparoscopic instruments, ergonomic design proposals have been developed in the handle, enhancing functionality in use and the surgeon’s comfort during Minimally Invasive Surgery (MIS) tasks. An example is the handle design for the laparoscopic instrument grasper presented by González et al., [9–11], which ergonomically adapts to 4 different sizes of surgeons’ hands, based on hand anthropometry and positioning. Another proposal is introduced by Sancibrian et al., [12], featuring an ergonomic handle that reduces high-pressure zones in the hand and uncomfortable wrist positions for the surgeon, enabling more intuitive manipulation. Tung et al., [13] tested a handle designed with pistol-type ergonomic considerations, allowing the surgeon’s hand to be positioned more neutrally and incorporating comprehensive actuation mechanisms for instrument control.

The objective of this study is to introduce a novel laparoscopic needle holder with an ergonomically designed handle that readily conforms to the surgeon’s palm, enhancing the direct transmission of wrist rotation movements to the tool tip in the intracorporeal suturing tasks, decreasing the muscular strain on the limb during usage. This study quantifies the impact and operational advantages of our ergonomic laparoscopic needle holder in comparison to a conventional laparoscopic needle holder with straight handle, specifically in the context of intracorporeal suture knotting procedures.

## 2. Materials and methods

### 2.1. Requirements

**Fig 1:**
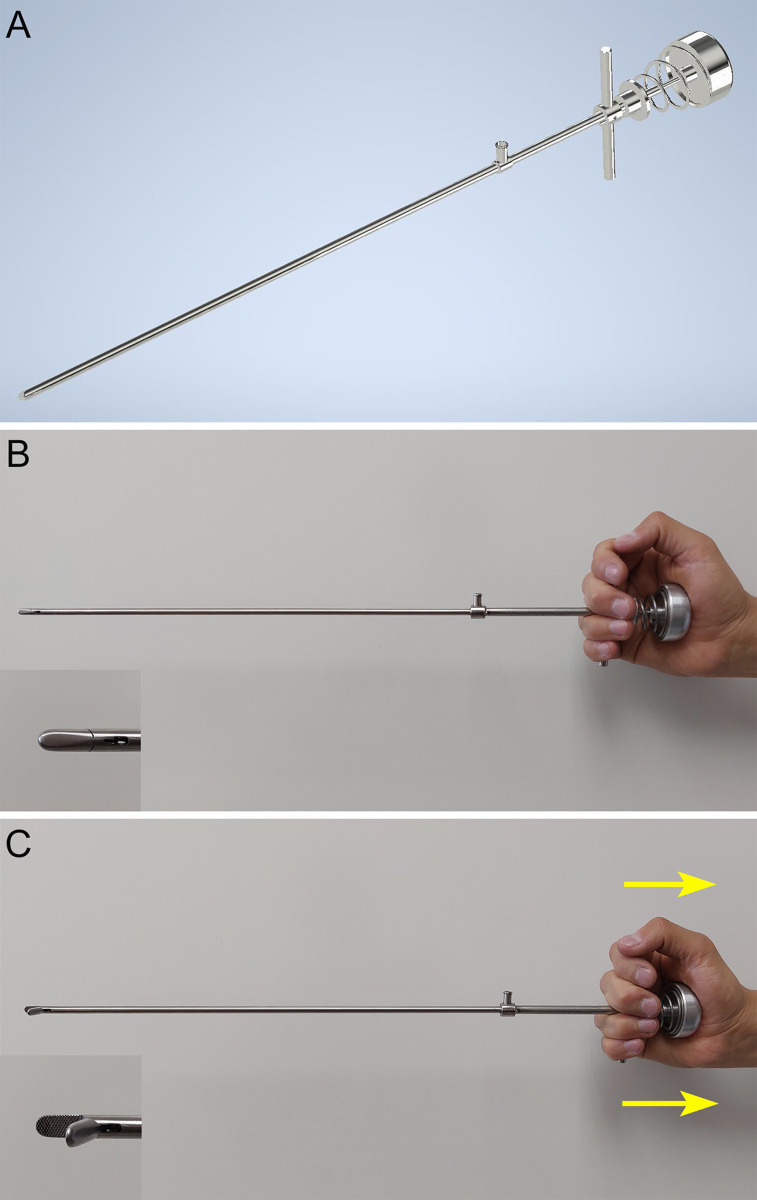
A Laparoscopic needle driver with ergonomic handle. B Instrument tip normally closed with semisphere and spring at rest. C Tip activation through spring compression with the semisphere.

The design of the laparoscopic needle holder with an ergonomic handle was carried out in close collaboration with the surgeons from the Pediatric Surgery Department of the Hospital Infantil de México Federico Gómez in Mexico City. This collaboration established the requirements for developing the new handle for this instrument. During these meetings with experienced laparoscopy surgeons, advantages and disadvantages in handling and operating the conventional laparoscopic needle holder were identified, leading to the establishment of design requirements and criteria for modifying the handle. This ergonomic handle was designed to be operated in a natural and straight position by the hand, allowing intuitive control of the rotation of the tip on its own axis with the surgeon’s wrist, as well as optimizing the opening and closing mechanism of the instrument (Fig 1A). The handle consists of a semi-spherical element that ergonomically adapts to the surgeon’s palm during use, enabling the opening (Fig 1B) and closing (Fig 1C) of the tip by compressing a spring using the fingers while maintaining the tip of this instrument in a normally closed configuration. The compression of the spring, which increases or decreases the pressure for gripping the suture needle with the laparoscopic needle holder’s tip, can be manually adjusted by rotating a threaded ring/support. This instrument design with an ergonomic handle was selected by the surgeons as the best concept because it maintains simplicity in its operation for opening and closing the tip and placing the suture needle in the jaws. Additionally, it allows a comfortable position for the wrist and along the same axis of action of the instrument for manual rotation of the end effector.

For the use of the ergonomic laparoscopic needle holder, the instrument should be held in the half-closed hand of a relaxed manner, placing the fingers on the grip. From this position, precise control of the instrument is obtained, which allows the direct transfer of rotational movements within a neutral angle of the wrist, turning the tool axis into an extension of the rotation axis of the surgeon’s forearm. To open the tip, the surgeon must apply a compressive force to the semi-spherical handle with his palm and supported by his fingers, very similar to the amount of force applied to a conventional laparoscopic needle holder with its ratchet mechanism to close the tip. Once the needle is placed close to the jaws, the surgeon should release the semi-spherical handle with his palm and fingers so that the tip of the instrument closes and firmly grips the suture needle without shifting during the task. In this way, the instrument allows the needle to be safely driven in the workspace, without releasing the needle from the tip to introduce it through the organs and tissues without rotating the needle. The mechanism in the ergonomic handle, activated by a spring, allows you to choose the appropriate pressure of the jaws through the adjustment of the threaded ring/support to hold the different types of suture needles firmly without damaging any of them.

In general, the laparoscopic needle holder with ergonomic handle does not require the surgeon to constantly apply force to open and close the tip during suturing tasks. This instrument allows you to control the force exerted, reduce excessive hand compressing that could cause tissue damage, as well as reduce the risks of fatigue in the shoulder and forearm.

### 2.2. Participants

The study was conducted at the Surgical Skills Laboratory of the General Surgery Department of the hospital. This study involved 15 right-handed pediatric surgery residents, ranging from PGY-3 to PGY-4, with experience in less than 10 performed laparoscopic procedures, and 7 experienced right-handed surgeons with a background of over 100 performed laparoscopic procedures. This study started with the recruitment of participants on 10/07/2023, closing registration on 17/07/2023. At the time of registration, written informed consent was obtained from all participants. Additionally, each participant was requested to complete a brief questionnaire outlining their demographic information and age. The research protocol for this study was approved by the Ethics, Research and Biosafety Committee of the Faculty of Medicine of the UNAM under protocol number **FM/DI/001/2023**.

### 2.3. Study design

This study required all participants to conduct an intracorporeal suturing task using the *EndoViS* laparoscopic simulator. This simulator is equipped with a video-based tracking system designed for the capture and recording of the 3D movement of laparoscopic instruments at a resolution of 0.14 mm at an acquisition rate of 30 frames per second (fps) using color markers (green and blue) and computer vision algorithms. With four entry ports, this laparoscopic simulator offers the capability for instrument insertion and provides a 0° view on an external monitor through a 750TVL color mini-camera [14, 15]. Within the *EndoViS* simulator, the model of the intracorporeal suturing task, based on the MISTELS protocol [16] and the FLS program [17, 18], was placed. This task consisted of grasping the suture needle with a laparoscopic needle holder, puncturing, and knotting a silk suture through two pre-marked points in a longitudinally slit Penrose drain. Then, the suture was tied using an intracorporeal knot technique. For this study, a thread length of 12 cm of 2–0 silk suture on a 26 mm taper needle was used. The initial and final position of instruments was indicated by two perforated holes in the plastic base of the exercise. To provide the same condition for all participants, the task’s position inside the simulator, the instruments used, the position and angle of the laparoscope showing the same image on the external monitor and the entry ports in the simulator were all standardized for each of them. Additionally, the workstation, where the *EndoViS* laparoscopic simulator was placed, was adjusted to the suitable height and position for each participant during the trials.

**Fig 2:**
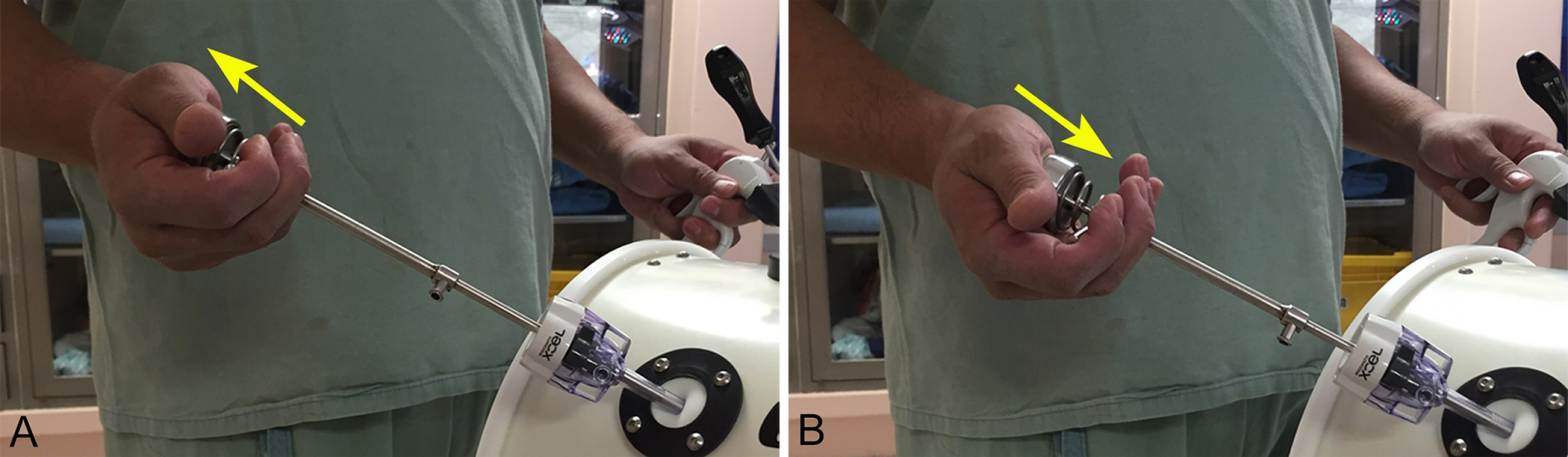
Testing setup for the study of the laparoscopic needle driver with ergonomic handle. A) Semisphere compression with palm and fingers for opening the jaws. B) Semisphere releasing for jaws closing and grasping the needle with the instrument.

The study was conducted across two consecutive sessions. In the initial session, participants standardized the procedure to performing intracorporeal suturing tasks employing the knot-tying technique. Subsequently, they executed this task thrice using two conventional laparoscopic needle holders with straight handles, recording the laparoscopic instruments movements during their final attempt. In this session, pediatric surgeons and residents were introduced to the modified instrument, the laparoscopic needle holder with ergonomic handle, and were given the opportunity to acquaint themselves with the tool. All participants were instructed on the proper method of holding the surgical instrument in hand and how to open and close the tip jaws by compressing the spring with the fingers and palm of the hand (Fig 2A and 2B), due to the normally closed configuration of the instrument, for a maximum of 5 minutes without performing the suture task and outside the simulator. In the second session, participants performed the same intracorporeal suturing task three times using the laparoscopic needle holder with ergonomic handle in their dominant hand and a laparoscopic needle holder with straight handle in their non-dominant hand. For this study, the movement of the laparoscopic instruments was recorded during the final attempt. Throughout all the study, no time limit was imposed for completing each of the three intracorporeal sutures for performance analysis using the two laparoscopic needle holders. Additionally, the Penrose drain tube was replaced for each participant to prevent prior stitches from limiting or influencing the subsequent participants’ performance.

### 2.4. Assessment of the performance

Kinematic motion data from the two instruments, with the straight and ergonomic handle, in the participants’ dominant hand were captured and analyzed using 16 motion analysis parameters (MAPs) [14, 19, 20], Table 1. All MAPs were calculated from the position *x* = (*x*,*y*,*z*) of laparoscopic instruments derived from the video-based tracking system integrated within the *EndoViS* simulator and computed using MATLAB Release 2022b (MathWorks, Natick, MA). For this study, the MAPs values obtained from the laparoscopic needle holders in the dominant hand were used to compare the performance of the participants with both instruments. The S1 File shows the calculated scores of the 16 MAPs for the ergonomic handle, while the S2 File shows the calculated values for the conventional straight handle.

**Table 1:** Description MAP’s for assessing the performance in intracorporeal suturing.

MAPs	Definition	Equation
**Time**	The total time required to perform the task(s). (seg)	T
**Bimanual Dexterity**	The correlation between the velocities of both instruments during the task(s). (-)	$\frac{\sum_{n=1}^{N}\left(v_{left}(n)-{\bar{v}}_{left})(v_{right}(n)-{\bar{v}}_{rigth}\right)}{\sqrt{\sum_{n=1}^{N}{\left(v_{left}(n)-{\bar{v}}_{left}\right)}^{2}\sum_{n=1}^{N}{\left(v_{right}(n)-{\bar{v}}_{rigth}\right)}^{2}}}$
**Path Length**	Total path followed by the tip of the instrument while performing the task(s). (m)	$\int_{t=0}^{T}\sqrt{{\left(\frac{dx}{dt}\right)}^{2}+{\left(\frac{dy}{dt}\right)}^{2}+{\left(\frac{dz}{dt}\right)}^{2}dt}$
**Depth Perception**	Total distance travelled by the instrument along its axis. (m)	$\int_{t=0}^{T}\sqrt{{\left(\frac{dy}{dt}\right)}^{2}+{\left(\frac{dz}{dt}\right)}^{2}dt}$
**Depth along Trocar**	Total distance travelled by the instrument with respect to the trocar’s coordinate. (m)	$D_{Tr}=\sqrt{{\left(x_{Tr}-x_{n}\right)}^{2}+{\left(y_{Tr}-y_{n}\right)}^{2}+{\left(z_{Tr}-z_{n}\right)}^{2}}$
		$\int_{t=0}^{T}\frac{dD_{Tr}}{dt}dt$
**Motion Smoothness**	Abrupt changes in acceleration resulting in jerky movements of the instrument. (m/s^3^)	$MS=\frac{J}{T},\;J=\sqrt{\frac{1}{2}\int_{t=0}^{T}{\left({\left(\frac{d^{3}x}{{dt}^{3}}\right)}^{2}+{\left(\frac{d^{3}y}{{dt}^{3}}\right)}^{2}+{\left(\frac{d^{3}z}{{dt}^{3}}\right)}^{2}\right)}^{2}dt}$
**Average**	Rate of change of the position of the instrument. (mm/s)	$\frac{1}{T}\int_{t=0}^{T}\sqrt{{\left(\frac{dx}{dt}\right)}^{2}+{\left(\frac{dy}{dt}\right)}^{2}+{\left(\frac{dz}{dt}\right)}^{2}dt}$
**Velocity**		
**Average Acceleration**	Rate of change of the velocity of the instrument. (mm/s^2^)	$\frac{1}{T}\int_{t=0}^{T}\sqrt{{\left(\frac{d^{2}x}{{dt}^{2}}\right)}^{2}+{\left(\frac{d^{2}y}{{dt}^{2}}\right)}^{2}+{\left(\frac{d^{2}z}{{dt}^{2}}\right)}^{2}dt}$
**Idle time**	Percentage of time where the instrument was considered still. (%)	$\frac{\left|I\right|}{T}:I=\frac{1}{T}\int_{t=0}^{T}\sqrt{{\left(\frac{dx}{dt}\right)}^{2}+{\left(\frac{dy}{dt}\right)}^{2}+{\left(\frac{dz}{dt}\right)}^{2}dt}\leq 5$
**Economy of area (EOA)**	Relation between the maximum surface area covered by the instrument and the total path. (-)	$\frac{\sqrt{\left[Max(x)-Min(x)]∙[Max(y)-Min(y)\right]}}{PL}$
**Economy of volume (EOV)**	Relation between the maximum volume covered by the instrument and the total path. (-)	$\frac{\sqrt[{3}]{\left[Max(x)-Min(x)]∙[Max(y)-Min(y)]∙[Max(z)-Min(z)\right]}}{PL}$
**Energy of Area**	Energy inverted by the instrument over the surface area covered. (J/cm^2^)	$\frac{\sum_{t=0}^{T}{\left|x_{i}\right|}^{2}+\sum_{t=0}^{T}{\left|y_{i}\right|}^{2}}{\left[Max(x)-Min(x)]∙[Max(y)-Min(y)\right]}$
**Energy of Volume**	Energy inverted by the instrument over the volume covered. (J/cm^3^)	$\frac{\sum_{t=0}^{T}{\left|x_{i}\right|}^{2}+\sum_{t=0}^{T}{\left|y_{i}\right|}^{2}+\sum_{t=0}^{T}{\left|z_{i}\right|}^{2}}{\left[Max(x)-Min(x)]∙[Max(y)-Min(y)]∙[Max(z)-Min(z)\right]}$
**Angular Length**	Total change in angular position of the instrument. (degrees)	$AL=\int_{0}^{T}\sqrt{{\left(\frac{d\alpha }{dt}\right)}^{2}+{\left(\frac{d\beta }{dt}\right)}^{2}}dt,\alpha =\cos^{-1}\left(\frac{x}{\sqrt{x^{2}+y^{2}+z^{2}}}\right),\beta =\cos^{-1}\left(\frac{y}{\sqrt{x^{2}+y^{2}+z^{2}}}\right)$
**Response Orientation**	The total amount of instrument rotation around its axis. (degrees)	$RO=\int_{0}^{T}\sqrt{\left|\frac{d\gamma }{dt}\right|}dt,\gamma =\cos^{-1}\left(\frac{z}{\sqrt{x^{2}+y^{2}+z^{2}}}\right)$
**Number of Submovements**	Movement of the instrument that contains a speed larger than 15 mm/s. (-)	$NoS=\sum_{i=0}^{n}submovement$,
		Where **n** is the length of the speed array and:
		$submovement=\left\{ \begin{array}{l} 1,\;speed(i)>15\\ 0,\;otherwise \end{array}\right.$

### 2.5. Statistics analysis

Data was analyzed using SPSS software for Windows (SPSS Inc., Chicago, USA). Shapiro-Wilk test was used to determine the normality level of the data obtained. Nonparametric test *U* of Mann-Whitney was used to compare the surgeons and student’s performance using both laparoscopic needle drivers. A value of *p*≤0.05 was considered statistically significant.

## 3. Results

A total of 22 participants were included in this study: 15 pediatric surgery residents (mean age 25.29 ± 2.17 years; comprising 6 females and 9 males) and 7 expert male surgeons (mean age 41 ± 9.78 years). The group of Experts consisted of active pediatric surgeons in general surgery, urology, and neonatal surgery services at the hospital. All participants completed both sessions for this study. The results of the performance of Expert and Resident groups using the conventional laparoscopic needle holder and the ergonomic laparoscopic needle holder in their dominant hand are presented in Table 2. The overall performance of all 22 participants using both instruments is showed in Table 3.

**Table 2:** Results of the participants’ performance with both laparoscopic needle drivers. For all MAP’s mean scores, standard deviation and *p* values are given.

	Experts (n = 7)			Residents (n = 15)		
MAPs	Conventional	Ergonomic	*p* ^*a*^	Conventional	Ergonomic	*p* ^*a*^
**Time (s)**	102.22 ± 16.50	95.27 ± 16.74	0.247	303.60 ± 69.41	238.61 ± 71.33	**<0.001**
**Bimanual dexterity (-)**	0.62 ± 0.12	0.56 ± 0.16	0.216	0.40 ± 0.13	0.39 ± 0.17	0.822
**Path Length (m)**	1.69 ± 0.27	1.83 ± 0.30	0.226	6.09 ± 2.05	4.58 ± 1.66	**0.003**
**Depth Perception (m)**	1.32 ± 0.22	1.45 ± 0.27	0.229	4.84 ± 1.57	3.62 ± 1.27	**0.003**
**Depth along Trocar (m)**	0.86 ± 0.17	0.96 ± 0.19	0.193	3.17 ± 0.97	2.38 ± 0.85	**0.004**
**Motion Smoothness (m/s** ^**3**^ **)**	3135.51 ± 1043.17	3357.93 ± 1035.39	0.945	6828.54 ± 1686.56	5280.13 ± 1311.81	**0.004**
**Average Speed (mm/s)**	9.17 ± 1.39	10.29 ± 1.60	0.092	9.52 ± 1.71	9.64 ± 1.62	0.861
**Average Acceleration (mm/s** ^**2**^ **)**	12.64 ± 2.45	14.51 ± 1.80	0.084	13.43 ± 2.42	13.55 ± 2.48	0.907
**Idle Time (%)**	40.07 ± 5.56	32.88 ± 7.09	0.109	33.97 ± 6.93	34.14 ± 9.13	0.960
**EOA (-)**	0.04 ± 0.01	0.03 ± 0.01	**0.011**	0.01 ± 0.01	0.01 ± 0.01	**0.002**
**EOV (-)**	0.03 ± 0.01	0.03 ± 0.01	0.083	0.01 ± 0.01	0.01 ± 0.01	**0.001**
**Energy of Area (J/cm** ^**2**^ **)**	14.32 ± 5.69	18.03 ± 15.45	0.843	36.71 ± 15.70	30.85 ± 8.29	0.202
**Energy of Volume (J/cm** ^**3**^ **)**	738.41 ± 441.05	881.72 ± 845.29	0.640	1291.26 ± 596.35	1107.36 ± 323.97	0.301
**Angular Length (degrees)**	641.41 ± 127.04	660.89 ± 106.02	0.712	2079.62 ± 547.08	1549.17 ± 598.87	**0.001**
**Response Orientation (degrees)**	30.28 ± 7.40	30.86 ± 5.15	0.774	100.92 ± 21.69	76.18 ± 26.59	**<0.001**
**Number of Submovements (-)**	31.00 ± 6.39	33.12 ± 5.96	0.404	108.60 ± 38.32	84.06 ± 37.69	**0.036**

^***a***^ Mann–Whitney *U* test for analysis of differences between conventional and ergonomic laparoscopic needle drivers; significant at *p* ≤ 0.05 bold.

**Table 3:** Participants’ performance with both laparoscopic needle drivers. For all MAP’s, mean scores, standard deviation, and *p* values are given.

	Laparoscopic needle drivers		
MAPs	Conventional	Ergonomic	*p* ^*a*^
	(*n* = 22)	(*n* = 22)	
**Time (s)**	233.55 ± 113.00	188.75 ± 90.55	**<0.001**
**Bimanual dexterity (-)**	0.48 ± 0.16	0.45 ± 0.18	0.418
**Path Length (m)**	4.56 ± 2.70	3.63 ± 1.89	**0.012**
**Depth Perception (m)**	3.61 ± 2.13	2.86 ± 1.47	**0.016**
**Depth along Trocar (m)**	2.36 ± 1.36	1.88 ± 0.97	**0.032**
**Motion Smoothness (m/s** ^**3**^ **)**	5544.01 ± 2321.81	4611.54 ± 1520.67	**0.023**
**Average Speed (mm/s)**	9.40 ± 1.58	9.87 ± 1.61	0.350
**Average Acceleration (mm/s** ^**2**^ **)**	13.15 ± 2.40	13.88 ± 2.27	0.328
**Idle Time (%)**	36.09 ± 7.02	33.71 ± 8.33	0.340
**EOA (-)**	0.02 ± 0.01	0.02 ± 0.01	0.622
**EOV (-)**	0.01 ± 0.01	0.02 ± 0.01	0.189
**Energy of Area (J/cm** ^**2**^ **)**	28.92 ± 16.91	26.39 ± 12.59	0.622
**Energy of Volume (J/cm** ^**3**^ **)**	1098.97 ± 600.58	1028.87 ± 553.36	0.776
**Angular Length (degrees)**	1579.37 ± 828.33	1240.21 ± 647.24	**0.033**
**Response Orientation (degrees)**	76.35 ± 38.73	60.42 ± 30.75	**0.001**
**Number of Submovements (-)**	81.60 ± 48.74	66.34 ± 39.12	0.115

^***a***^ Mann–Whitney *U* test for analysis of differences between conventional and ergonomic laparoscopic needle drivers; significant at *p* ≤ 0.05 bold.

Significant statistical differences were observed in the group of residents in 10 of the 16 MAPs, including time, path length, depth perception, depth along trocar, motion smoothness, economy of area, economy of volume, angular length, response orientation, and number of submovements. Regarding the performance of the Expert group, statistically significant difference was found in the parameter of economy of area.

In the performance analysis of all study participants, Residents and Experts (n = 22), statistically significant differences were found in 7 evaluated MAPs. Overall, participants showed improvement in laparoscopic psychomotor skills, as well as in the ability to control the angular position and rotation of the instrument with respect to its own axis in the comparison between both laparoscopic needle holders, achieving significant differences in the angular length and response orientation parameters.

## 4. Discussion

The ability to perform intracorporeal suturing with an acceptable level of skill is an essential component in advanced laparoscopic surgery. Suturing technique involving intracorporeal knot tying is regarded as a difficult and complex skill for surgeons to acquire during their training. Nonetheless, mastering this technique is vital for the development of a wide range of technically demanding advanced laparoscopic procedures.

This study introduces the development and preliminary validation of a laparoscopic needle holder with an ergonomic handle design of 5mm diameter and overall length of 465mm, which enhances the capabilities and improves the MIS performance of the surgeon, facilitating the execution of intracorporeal knot-tying tasks in laparoscopic procedures. Due to its ergonomic design, the surgeon’s hand can be aligned with the main axis of the instrument, enabling pronation and supination movements of the wrist to produce a direct and continuous rotation around the tool’s axis. Also, with its normally closed configuration, the surgeon expends less effort to open and close the instrument, reducing muscular strain in the limb and allowing a focus on the tissue rather than the manipulation of the tip during its use in suturing tasks.

In addition, this ergonomic laparoscopic needle holder is designed to fit both the dominant and non-dominant hand, with the advantage of comfortably adjusting to the different hand sizes of surgeons, a feature observed during the validation testing stage of the instrument. The construction and assembly of this ergonomic laparoscopic needle holder were carried out using entirely market-available mechanical components. This approach facilitated the creation of a functional, sterilizable, and reusable instrument, making it suitable for future utilization in the operating room as part of the laparoscopic tool set.

Previously, three prototypes of this ergonomic laparoscopic needle holder were manufactured before arriving at the final design shown in this study. In the first version, the main concept of our tool was developed, with the semi-spherical handle, a straight spring to open and close the tip in the normally closed configuration, and finger grips. In this version, it was observed that the jaws did not have enough pressure to keep the needles without displacing or rotating. Therefore, the threaded ring/support was included to adjust the clamping force of the needles on the jaws. In the second version, the diameter and length of the finger grips were modified to improve the distribution of force for the opening and closing of the tip. In the latest version, the straight spring was replaced with a conical spring and the size of the semi-spherical handle diameter was modified to improve the distribution of compression force that is made with palm to open and close the tip in a more extended area of the hand.

In this study, the Residents group demonstrated an improvement in their performance in intracorporeal suturing tasks using the ergonomic laparoscopic needle holder compared to the Experts group, with statistically significant differences observed in 10 performance metrics (MAPs). This group of trainees showed optimization in task execution time, as well as in the kinematics and economy of instrument movement within the workspace during the suturing task with this proposed tool. Furthermore, they achieved significant improvements in aspects related to changes in angular position and instrument rotation, as well as a reduction in the number of abrupt movements performed during the exercise. These results demonstrate the advantages of this ergonomic design over the conventional instrument for surgeons in training. On the other hand, the Experts group demonstrated the optimization of the surgical workspace during the suturing task using this ergonomic laparoscopic needle holder in this study.

In general, the results from all participants indicated that the laparoscopic needle holder with ergonomic handle outperformed the laparoscopic needle holder with straight handle, particularly concerning parameters analyzing tip movement, angular position, and self-rotation of the instrument. Specifically, a reduction in the parameters of path length, depth perception, depth along trocar, and motion smoothness was observed, averaging approximately 19.58%. We attribute this reduction in kinematic scores to the intuitive control provided by the ergonomic interface of the laparoscopic needle holder for the manipulation and operation of the instrument tip. The ergonomic design of the handle, based on a semi-spherical mechanism with spring activation, maintains an optimal diameter that accommodates different hand sizes among participants. Additionally, it facilitates the positioning of the surgeon’s hand and forearm through the lateral entry ports. Moreover, the activation of the jaws, set to normally closed, is an enhancement that reduces excessive effort required to open and close the instrument tip during suturing tasks, thereby decreasing motor strain from the shoulder to the forearm during use. The threaded adjustment ring for the spring allows for the selection of appropriate pressure for the instrument jaws when using different surgical needles, preventing them from slipping off the tip or becoming damaged. On the other hand, a reduction in scores was also observed in the angular length and response orientation parameters, averaging 21.18%. We attribute this improvement to our design, which enables the transmission of axial rotational movements from the surgeon’s hand directly along the longitudinal axis of the instrument to the tip. This reduction in total rotations and angulations of the tool during handling allowed participants to focus more on the task and the necessary maneuvers for performing intracorporeal knot-tying suturing techniques.

In the literature, several studies have tried to improve ergonomic conditions by redesigning or modifying the handle of the laparoscopic instruments. Most of these proposals are similar to each other, which maintain the shape of a pistol grip in laparoscopic graspers [8–13]. In the ERGOLAP project, they designed an ergonomic handle in a laparoscopic grasper, which adapts to the different hand sizes of surgeons, and was evaluated by comparing its performance with a conventional laparoscopic grasper, through sEMG signals and Singular Spectrum Analysis (SSA), where demonstrated that its pistol grip reduces the mean values of the muscle activity [9–11]. The proposal by Sancibrian et al., [12] based on a pistol handle, was evaluated through a goniometric study of angular movement using electrodes for electromyography (EMG). The pistol grip design of Tung et al.,[13] was evaluated primarily by surgeon elapsed time and completion on FLS tasks and subjective user ratings. In their study, they reported that their proposed mechanism allows surgeons to have the hand in a more neutral position, as well as less pain in places such as arms and hands.

A similar handle design to ours is the LaproFlex laparoscopic instrument [21], which was evaluated of performance with expert surgeons and novices. This instrument was subjectively evaluated in basic suturing tasks, comparing it with straight laparoscopic instruments and the total time for the procedure. In our proposed, the ergonomic handle allows surgeons to keep the hand in a neutral posture and improving the activation mechanism of the tip because of the spring and the normally closed configuration. The semi-spherical element contacts all the palm hand and the index and ring fingers only must smoothly press against the spring to open and close the tip. This produces that the effort to activate the instrument is more distributed.

Some limitations of our study involved the sample size of 22 participants from the specialty of pediatric surgery. While the results of the MAPs showed significant differences in surgeons’ performance with the laparoscopic needle holder with ergonomic handle, a larger sample that includes a diverse range of participants from different specialties, i.e. general surgery, gynecology, and urology, would improve the robustness of this study and the validation of this innovation. Also, participants with varying levels of laparoscopic experience such as novices, intermediates, and experts would provide a clearer and more complete understanding of the impact and skill level achieved with the ergonomic laparoscopic needle holder.

Another limitation is the level of laparoscopic competence of the participants. In this study, the laparoscopic needle holder with ergonomic handle was validated using different levels of laparoscopic experience, classified as Resident and Expert. Although the same level of competence of the participants is needed to obtain highest internal validity in the study, we found it interesting to demonstrate that the group of Residents were the most benefited by the ergonomic handle and its linear position of their hand and palm with the axis of the main of the instrument, in particular in metrics related to angular position and axial rotation, as well as the way to open and close the tip, which decreased the amount of its abrupt movements made during the suture task. However, in future studies we will use a similar level of laparoscopic surgical competence so that the validation study has high internal validity.

Another limitation of the study is the evaluation of the ergonomic laparoscopic needle holder, based on objective measures of performance through MAPs. To round off this study, the qualitative opinion and satisfaction of the surgeons on the comfort, usability and experience would further enrich the analysis of the ergonomics of the proposed laparoscopic needle holder. Future research will be conducted to improve this study and understand the comfort level, ease of use of the instrument in the face of any challenges faced by the surgeon during intracorporeal suturing.

For future surgical applications, it is necessary to implement a locking function to prevent uncomfortable needle positions or displacements when applying force to tissue during puncture. While the threaded ring allows for spring pressure adjustment for gripping different needles at the tip, a locking mechanism will provide additional security and confidence to surgeons when handling the instrument with the needle during sutures. The findings of this study revealed that the new handle design offers significant usability advantages for laparoscopic needle holders by economizing movement, reducing excessive muscle contraction, and providing a more natural position for the shoulder, forearm, and hand. Future studies will investigate how the new design of the ergonomic laparoscopic needle holder function in the hands of more experienced surgeons during training for laparoscopic end-to-end anastomoses using simulation models and *ex-vivo* tissue. Also, a long-term evaluation to investigate the effects of using the ergonomic laparoscopic needle holder and its impact on muscle fatigue and overall surgeon satisfaction over extended periods. Furthermore, we will incorporate electromyographic analysis of the involved limbs, along with the measurement of the physical and mental workload of surgeons during the utilization of this ergonomic laparoscopic needle holder in these studies.

## 5. Conclusion

In this study, we introduce an innovative laparoscopic needle holder with ergonomic handle designed for intracorporeal suturing procedures in laparoscopic surgery. This ergonomic laparoscopic needle holder outperformed the conventional laparoscopic needle holder in parameters assessing movement, angle, and rotation around the axis during its use in suturing tasks. The results indicated that this ergonomic handle design enhances the transmission of rotational movements from the surgeon’s hand to the longitudinal axis towards the instrument tip, reducing excessive efforts and muscle contraction in the forearm and hand when opening and closing the jaws. The design concept of the ergonomic laparoscopic needle holder allows for enhanced tool manipulation by improving the transfer of axial rotational movements from the surgeon’s hand directly to the main axis and tip, as well as providing comfort in the palm for use in intracorporeal laparoscopic sutures. Due to its intuitive operation, the laparoscopic needle holder with ergonomic handle is poised for future integration with surgeons in the operating room.

## Supporting information

S1 File MAPs calculated from participant performance using the ergonomic laparoscopic needle holder. (CSV)

S2 File MAPs calculated from participant performance using the conventional laparoscopic needle holder. (CSV)
